# Scaling the side effects: A case of apalutamide-induced ichthyosis

**DOI:** 10.1016/j.jdcr.2025.06.001

**Published:** 2025-06-18

**Authors:** Aneri B. Patel, Christine T. Pham, Janellen Smith

**Affiliations:** a Department of Dermatology, University of California, Irvine, CA; b School of Medicine, University of California, Davis, Sacramento, CA

**Keywords:** acquired ichthyosis, apalutamide, apalutamide-induced ichthyosis, ichthyosis

## Introduction

Ichthyosis refers to a diverse group of inherited or acquired skin conditions marked by excessive production of the corneal layer, causing dry, scaly skin. Acquired ichthyosis (AI) may be a result of malignancy (Hodgkin’s, multiple myeloma), nutritional diseases (celiac disease, pancreatic insufficiency), metabolic disease (chronic hepatic or renal failure), infectious disease (acquired immunodeficiency syndrome, leprosy), or the use of certain medications, like cimetidine, cholesterol-lowering agents, and hydroxyurea.^1^

Apalutamide is a novel antiandrogen medication approved in the United States in 2018 based on the results of Selective Prostate Androgen Receptor Targeting with ARN-509 (former name of apalutamide), a multicenter, placebo-controlled, randomized phase 3 clinical trial in men with nonmetastatic castration-resistant prostate cancer.^2^ Its mechanism of action is selective binding to the ligand domain of the androgen receptor (AR), thereby inhibiting receptor translocation to the nucleus and its transcriptional activity.^2^ The most common adverse reactions associated with this medication include fatigue, hypertension, skin-related issues, diarrhea, and nausea. Twenty-six percent of patients have a skin reaction. These include pruritus, exanthematous erutpions, butterfly rash, erythema multiforme, lichenoid rash, urticaria, xerosis, and eczematous eruptions.^3^^,^^4^ More severe conditions like acute generalized exanthematous pustulosis, toxic epidermal necrolysis, and Stevens-Johnson syndrome have been rarely documented.^2^ Of note, there has only been 1 case reported of AI due to this medication.^3^ Here, we report a second case, further adding to the literature of this rare association.

## Case report

A 76-year-old man with high-risk prostate cancer (cT2bN0M0, Gleason 4+5) status post definitive radiotherapy presented to dermatology with a 3-month history of a pruritic, erythematous eruption involving his entire body. This macular rash initially appeared on his thighs 2 weeks after initiating treatment with relugolix (120 mg daily) and apalutamide (240 mg daily). Radiation oncology suspected a grade 1 reaction to apalutamide and initiated treatment with diphenhydramine hydrochloride 1% cream, hydrocortisone 2.5% cream, and the erythema improved over a month. However, in another month, he developed lower extremity swelling and a pruritic rash. The patient stopped taking apalutamide but continued taking relugolix and was referred to dermatology. He also reported a burning sensation beneath the skin but denied any mucosal involvement or oral lesions. The patient had no personal or family history of ichthyosis or atopy. Apart from relugolix and apalutamide, no new medications had been introduced.

**Fig 1:**
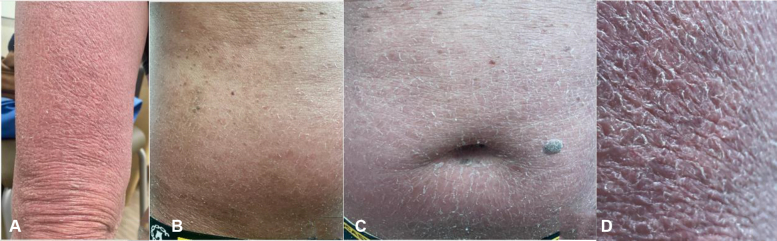
**A-D,** Fine, white scale appearing in a fish-scale pattern along with background erythema present on the bilateral legs **(A)**, trunk **(B)**, and around the umbilicus **(C)**. **D**, Close-up image of the scale with some underlying hyperpigmentation of the skin.

Physical examination showed ill-defined erythema on the lower trunk extending through the buttocks and bilateral lower extremities with an overlying ichthyosiform scale, accompanied by 1+ pitting edema on the lower extremities (Fig 1, *A*-*E*). Dermatoscopic examination showcased fine white scales forming prominent, crisscross patterns across the skin. No telangiectatic/dotted vessels were present.

The differential diagnosis included a paraneoplastic ichthyosiform eruption associated with prostate cancer and apalutamide-induced AI. Although there are no guidelines for diagnosing apalutamide-induced ichthyosis, given the temporal relationship, clinical presentation, morphology of the lesions, and reported association, apalutamide-induced AI was favored. The clear dermatologic presentation of adult-onset symmetric fine scaling without palmoplantar or mucosal involvement was consistent with AI, which is a clinical diagnosis. In such cases, biopsies are reserved for atypical or diagnostically ambiguous presentations. Laboratory investigations were not performed, as the patient had no systemic symptoms and no personal/family history of ichthyosis. The clear temporal association with apalutamide and absence of clinical features suggesting an alternative etiology supported a clinical diagnosis without further testing.

He was prescribed fluocinolone acetonide topical oil 0.01% twice daily to the scalp and as needed to the body for itching and urea cream 20% daily for the body. In addition, he discontinued apalutamide while continuing relugolix and reported an overall improvement in pruritus since initial presentation. He had previously attempted triamcinolone 0.1% ointment without notable improvement. The AI was nonlife-threatening, the patient was managing it well, and the benefits of continuing apalutamide were deemed to outweigh the risks, so the decision was made to maintain therapy at the same dose.

At his most recent follow-up, the patient was comfortable, with significant improvement in his lesions, pruritus, and with reduced scale. On examination, only minimal ichthyosiform scaling remained on the lower abdomen, with sparse involvement of the lower back and bilateral lower legs and with no residual erythema or pitting edema. He continued to tolerate the ichthyosis well with topical therapy (fluocinolone solution and urea cream).

## Discussion

Cancer treatments are known to cause skin eruptions that can have significant effects on not only the patient’s quality of life but may also influence decisions regarding modification or cessation of treatment. As new medications become available, it becomes imperative to recognize side effects.

Apalutamide is a nonsteroidal, second-generation antiandrogen medication that exerts its effect by directly inhibiting AR at the ligand-binding domain. This inhibition prevents AR nuclear translocation and transcription.^5^ By disrupting AR signaling, apalutamide decreases tumor cell proliferation and promotes apoptosis, contributing to a reduction in tumor burden.

Androgens such as testosterone are known to have a role in skin homeostasis, including effects on skin moisture, thickness, and elasticity.^6^ As the central event in the pathogenesis of ichthyosis is the disruption of the epidermal barrier causing scaly dry spots, it can be postulated that decreased testosterone due to apalutamide affects the skin, disrupting the skin barrier and causing dryness through corneal layer desiccation, and subsequent ichthyosis. Additionally, sebum helps lubricate the skin, reducing friction. Multiple studies have associated elevated testosterone levels with heightened sebaceous gland activity in humans, frequently resulting in excessive sebum production^7^; the decrease in testosterone can reduce sebum production and exacerbate dry skin.

Ciolfi et al^3^ previously reported a case of apalutamide-induced ichthyosiform eruption in a 69-year-old man with prostate cancer. To the best of our knowledge, our case represents the second documented case about apalutamide-induced AI. The diagnosis of drug-induced AI was supported by the timing of symptom onset, resolution with drug discontinuation, and a high Naranjo score indicating a “highly probable” adverse drug reaction compared with being idiopathic.^8^ Further studies are needed to characterize the spectrum of cutaneous adverse effects associated with apalutamide, as early recognition and management improves patient outcomes and facilitates continued use of this life-prolonging therapy.

## Conflicts of interest

All of the authors report no conflicts of interests.
